# Supplementary material for: Patterns of Intron Gain and Loss in Fungi
Source: PLoS Biol. 2004 Nov 30;2(12):e422. doi: 10.1371/journal.pbio.0020422 (PMC532390; doi:10.1371/journal.pbio.0020422)
Supplement: Table S1 — Also available at http://genes.mit.edu/NielsenEtAl/. (4.3 MB ZIP). [file pbio.0020422.st001.zip › NielsenEtAl/html/28.html]

AN4222.1.NCU01827.1.MG06693.1.FG05999.1


```
 CLUSTAL W (1.82) Multiple Sequence Alignments - Introns Inserted


Sequence 1: NCU01827.1	135 aa
Sequence 2: FG05999.1	170 aa
Sequence 3: MG06693.1	149 aa
Sequence 4: AN4222.1	135 aa
Alignment Length: 184 aa
Number Identitical Residues: 98 aa
Alignment Score (without introns) 4280


MG06693.1 	MKF~LKVGRVAIITRGRFAGKK0DQAADIVASSPPHQVVIIQPVDSGNKLHPYGHAIVAG
NCU01827.1	MKF~LKTSRVCLVTRGRYAGKK0--------------VVIIQPVDNGSKTHPYGHAIVAG
FG05999.1 	MKF~LKVGRVAIITRGRYAGKK0--------------VVIIQPVDNGNKPHPFGHALVAG
AN4222.1  	MKF1MKVGRVAIITRGRYAGKK0--------------VVIVQPQDSGSKAHPFSYAIVAG
          	*** :*..**.::****:****               ***:** *.*.* **:.:*:***

MG06693.1 	IERYPLKITRRMSKTRQEKRNKIKPFVKVVNYNHLMPTRYTLELEGLKGSVTNDTFKEVS
NCU01827.1	IERYPSKITRRMSKTRQEKRSKVKPFIKVINYNHLMPTRYTLELEGLKGSVSAETFKEVS
FG05999.1 	IERYPSKITRRMSKTRQEKRNKIKPFIKVINYNHLMPTRYTLELEGLKGAVSNDTFKEVS
AN4222.1  	IERYPLKVTRRMGKKTVEKRSRIKPFIKVVNYNHLMPTRYTLELEGLKGTVTADTFKEVS
          	***** *:****.*.  ***.::***:**:*******************:*: :******

MG06693.1 	QREDAKKNVKKVLEERYTSGKNRWFFTPL--~-K1F----------~-------------
NCU01827.1	QREDAKKTVKKVLEERYTSGKNRWFFTPL--~-R1F----------~-------------
FG05999.1 	QREDAKKTVKKVLEERYTSGKNRWFFTPLST2NR~FSITVRELAGC1FLCYPFRFYMSMA
AN4222.1  	QREEAKKTIKKALEDRYTSGKNRWFFTPL--~-R1F----------~-------------
          	***:***.:**.**:**************    : *                        

MG06693.1 	---------
NCU01827.1	---------
FG05999.1 	KRRLELHIN
AN4222.1  	---------
          	
```
